# Supplementary material for: Isolation and Biochemical Characterization of Six Anaerobic Fungal Strains from Zoo Animal Feces
Source: Microorganisms. 2021 Aug 3;9(8):1655. doi: 10.3390/microorganisms9081655 (PMC8399178; doi:10.3390/microorganisms9081655)
Supplement: Supplementary file 1 [file microorganisms-09-01655-s001.zip › Supplementary captions.pdf]

Tab. S1: Percent identity of the D1-D2 LSU sequences of the isolated strains to the sequences of other AF. Sequences were aligned in MAFFT and manually curated in BioEdit.

Fig. S1: Phylogenetic affiliation of the Isolate PP313 to other *Caecomyces* and *Cyllamyces* based on the nucleotide sequences of the ITS1 from the ribosomal operon. Isolate G341 was used as an outgroup. MAFFT [43] was used for alignment and BioEdit [42] was used for manual curation of the sequences. The ML-tree was constructed through IQTREE [44] with the predicted model HKY+F+G4 and –bb 1000.

Tab. S2: Percent identity of the ITS sequences of Isolate PP313 available *Caecomyces* and *Cyllamyces* sequences. Sequences were aligned in MAFFT and manually curated in BioEdit.

Tab. S3: Residual galactose during growth of different strains on Lactose in g/l and mmol.

Tab. S4: Produced metabolites from G341 while growing on different c-sources including standard deviation (SD) and excluding ethanol.

Tab. S5: Relative produced metabolites from G341 while growing on different c-sources including standard deviation (SD) and excluding ethanol.

Tab. S6: Produced metabolites from PP313 while growing on different c-sources including standard deviation (SD) and excluding ethanol.

Tab. S7: Relative produced metabolites from PP313 while growing on different c-sources including standard deviation (SD) and excluding ethanol.

Tab. S8: Produced metabolites from W212 while growing on different c-sources including standard deviation (SD) and excluding ethanol.

Tab. S9: Relative produced metabolites from W212 while growing on different c-sources including standard deviation (SD) and excluding ethanol.

Tab. S10: Produced metabolites from SA222 while growing on different c-sources including standard deviation (SD) and excluding ethanol.

Tab. S11: Relative produced metabolites from SA222 while growing on different c-sources including standard deviation (SD) and excluding ethanol.

Tab. S12: Produced metabolites from X2152 while growing on different c-sources including standard deviation (SD) and excluding ethanol.

Tab. S13: Relative produced metabolites from X2152 while growing on different c-sources including standard deviation (SD) and excluding ethanol.

Tab. S14: Produced metabolites from A252 while growing on different c-sources including standard deviation (SD) and excluding ethanol.

Tab. S15: Relative produced metabolites from A252 while growing on different c-sources including standard deviation (SD) and excluding ethanol.
