# Supplementary material for: Isolation and Biochemical Characterization of Six Anaerobic Fungal Strains from Zoo Animal Feces
Source: Microorganisms. 2021 Aug 3;9(8):1655. doi: 10.3390/microorganisms9081655 (PMC8399178; doi:10.3390/microorganisms9081655)
Supplement: Supplementary file 1 [file microorganisms-09-01655-s001.zip › Tables S3-S13.pdf]

Table S3: Residual galactose during growth of different strains on Lactose in g/l and mmol.

	g/l					mmol				
Strain	1	2	3	Ø	SD	1	2	3	Ø	SD
G341	0,790	1,017	0,961	0,923	0,096	0,241	0,310	0,293	0,282	0,029
PP313	1,823	2,070	1,982	1,958	0,102	0,556	0,632	0,605	0,598	0,031
W212	1,982	2,008	2,067	2,019	0,035	0,605	0,613	0,631	0,616	0,011
SA222	1,928	1,981	1,868	1,926	0,046	0,589	0,605	0,570	0,588	0,014
X2152	1,501	1,501	0,999	1,334	0,237	0,458	0,458	0,305	0,407	0,072
A252	0,175	0,125	0,118	0,139	0,025	0,053	0,038	0,036	0,043	0,008

Tab. S4: Produced metabolites from G341 while growing on different c-sources including standard deviation (SD) and excluding ethanol.

	Formate Δ [mmol]	Acetate Δ [mmol]	Lactate Δ [mmol]	Succinate Δ [mmol]	Citrate Δ [mmol]	H2 Δ [mmol]
Cellobiose Ø	1,510	1,251	0,792	0,105	0,000	0,358
Cellobiose SD	0,082	0,067	0,041	0,003	0,000	0,047
Cellulose Ø	1,437	1,073	0,914	0,114	0,000	0,313
Cellulose SD	0,026	0,015	0,038	0,002	0,000	0,030
Fructose Ø	1,503	1,225	0,757	0,133	0,000	0,232
Fructose SD	0,103	0,114	0,071	0,008	0,000	0,007
Glucose Ø	1,398	1,174	0,725	0,126	0,000	0,312
Glucose SD	0,054	0,041	0,037	0,008	0,000	0,023
Inulin Ø	1,138	1,050	0,572	0,112	0,000	0,282
Inulin SD	0,127	0,167	0,102	0,009	0,000	0,032
Lactose Ø	0,607	0,415	0,058	0,000	0,000	0,220
Lactose SD	0,044	0,042	0,009	0,000	0,000	0,010
Maltose Ø	1,356	1,217	0,766	0,125	0,000	0,366
Maltose SD	0,043	0,034	0,064	0,001	0,000	0,007
Mannose Ø	1,309	0,991	0,498	0,108	0,000	0,360
Mannose SD	0,110	0,109	0,095	0,003	0,000	0,036
Pectin Ø	0,138	0,000	0,000	0,000	0,000	0,025
Pectin SD	0,004	0,000	0,000	0,000	0,000	0,005
Starch Ø	1,388	1,161	0,610	0,112	0,000	0,307
Starch SD	0,168	0,112	0,108	0,019	0,000	0,004
Stroh Ø	1,017	0,770	0,140	0,000	0,011	0,352
Stroh SD	0,032	0,053	0,038	0,000	0,000	0,011
Sucrose Ø	1,475	1,327	0,852	0,136	0,000	0,335
Sucrose SD	0,026	0,077	0,067	0,003	0,000	0,033
Trehalose Ø	0,000	0,000	0,000	0,000	0,000	0,008
Trehalose SD	0,000	0,000	0,000	0,000	0,000	0,003
Xylan Ø	1,144	0,848	0,264	0,096	0,064	0,211
Xylan SD	0,017	0,010	0,010	0,003	0,000	0,012
Xylose Ø	1,449	1,368	0,713	0,117	0,000	0,278
Xylose SD	0,063	0,180	0,016	0,003	0,000	0,016

Tab. S5: Relative produced metabolites from G341 while growing on different c-sources including standard deviation (SD) and excluding ethanol.

	Formate/total [%]	Acetate/total [%]	Lactate/total [%]	Succinate/total [%]	Citrate/total [%]	H2/total [%]
Cellobiose Ø	37,641	31,138	19,710	2,603	0,000	8,908
Cellobiose SD	2,678	1,124	0,642	0,046	0,000	1,062
Cellulose Ø	37,334	27,882	23,718	2,952	0,000	8,115
Cellulose SD	1,148	0,269	0,576	0,046	0,000	0,654
Fructose Ø	39,098	31,754	19,654	3,445	0,000	6,050
Fructose SD	2,952	1,885	1,502	0,196	0,000	0,379
Glucose Ø	37,411	31,432	19,411	3,380	0,000	8,366
Glucose SD	1,277	1,261	0,914	0,208	0,000	0,656
Inulin Ø	36,255	33,187	18,014	3,583	0,000	8,962
Inulin SD	1,134	0,785	0,814	0,307	0,000	0,230
Lactose Ø	46,703	31,874	4,444	0,000	0,000	16,979
Lactose SD	0,487	0,806	0,363	0,000	0,000	0,672
Maltose Ø	35,424	31,794	19,971	3,256	0,000	9,554
Maltose SD	0,598	0,270	1,051	0,123	0,000	0,361
Mannose Ø	40,201	30,348	15,094	3,328	0,000	11,029
Mannose SD	1,046	0,156	1,395	0,318	0,000	0,236
Pectin Ø	84,606	0,000	0,000	0,000	0,000	15,394
Pectin SD	2,213	0,000	0,000	0,000	0,000	2,213
Starch Ø	38,758	32,533	16,906	3,108	0,000	8,695
Starch SD	0,934	1,180	1,263	0,231	0,000	1,026
Stroh Ø	44,424	33,636	6,098	0,000	0,472	15,370
Stroh SD	1,078	2,408	1,629	0,000	0,005	0,634
Sucrose Ø	35,763	32,155	20,653	3,300	0,000	8,130
Sucrose SD	0,902	1,588	1,564	0,098	0,000	0,782
Trehalose Ø	0,000	0,000	0,000	0,000	0,000	100,000
Trehalose SD	0,000	0,000	0,000	0,000	0,000	0,000
Xylan Ø	43,546	32,290	10,063	3,638	2,425	8,038
Xylan SD	0,446	0,309	0,332	0,140	0,009	0,480
Xylose Ø	36,993	34,695	18,224	2,996	0,000	7,092
Xylose SD	1,036	2,291	0,942	0,242	0,000	0,095

Tab. S6: Produced metabolites from PP313 while growing on different c-sources including standard deviation (SD) and excluding ethanol.

	Formate Δ [mmol]	Acetate Δ [mmol]	Lactate Δ [mmol]	Succinate Δ [mmol]	Citrate Δ [mmol]	H2 Δ [mmol]
Cellobiose Ø	1,319	1,005	0,676	0,194	0,000	0,547
Cellobiose SD	0,043	0,024	0,074	0,002	0,000	0,026
Cellulose Ø	0,000	0,000	0,071	0,000	0,000	0,022
Cellulose SD	0,000	0,000	0,052	0,000	0,000	0,006
Fructose Ø	1,195	0,892	0,608	0,140	0,000	0,475
Fructose SD	0,119	0,072	0,069	0,011	0,000	0,047
Glucose Ø	1,165	0,647	0,711	0,156	0,000	0,488
Glucose SD	0,086	0,136	0,052	0,017	0,000	0,019
Inulin Ø						
Inulin SD						
Lactose Ø	0,850	0,703	0,143	0,106	0,000	0,344
Lactose SD	0,037	0,062	0,023	0,016	0,000	0,018
Maltose Ø	0,000	0,000	0,053	0,000	0,000	0,027
Maltose SD	0,000	0,000	0,038	0,000	0,000	0,002
Mannose Ø						
Mannose SD						
Pectin Ø	0,000	0,228	0,116	0,000	0,000	0,009
Pectin SD	0,000	0,162	0,007	0,000	0,000	0,003
Starch Ø	0,000	0,107	0,080	0,000	0,000	0,017
Starch SD	0,000	0,151	0,001	0,000	0,000	0,001
Stroh Ø	0,592	0,527	0,115	0,000	0,009	0,322
Stroh SD	0,004	0,007	0,009	0,000	0,006	0,008
Sucrose Ø						
Sucrose SD						
Trehalose Ø						
Trehalose SD						
Xylan Ø	1,098	0,891	0,310	0,160	0,046	0,337
Xylan SD	0,019	0,043	0,019	0,014	0,001	0,012
Xylose Ø	1,093	0,836	0,772	0,130	0,000	0,400
Xylose SD	0,068	0,056	0,157	0,017	0,000	0,008

Tab. S7: Relative produced metabolites from PP313 while growing on different c-sources including standard deviation (SD) and excluding ethanol.

	Formate/total [%]	Acetate/total [%]	Lactate/total [%]	Succinate/total [%]	Citrate/total [%]	H2/total [%]
Cellobiose Ø	35,251	26,875	18,036	5,177	0,000	14,661
Cellobiose SD	0,144	0,365	1,396	0,122	0,000	1,069
Cellulose Ø	0,000	0,000	56,384	0,000	0,000	43,616
Cellulose SD	0,000	0,000	40,128	0,000	0,000	40,128
Fructose Ø	36,033	26,932	18,402	4,226	0,000	14,406
Fructose SD	0,941	0,486	1,709	0,121	0,000	1,548
Glucose Ø	36,839	20,222	22,509	4,918	0,000	15,511
Glucose SD	0,794	2,503	0,828	0,322	0,000	1,067
Inulin Ø						
Inulin SD						
Lactose Ø	39,633	32,711	6,638	4,974	0,000	16,043
Lactose SD	0,559	1,265	0,795	0,977	0,000	0,102
Maltose Ø	0,000	0,000	49,627	0,000	0,000	50,373
Maltose SD	0,000	0,000	35,097	0,000	0,000	35,097
Mannose Ø						
Mannose SD						
Pectin Ø	0,000	49,338	46,641	0,000	0,000	4,020
Pectin SD	0,000	34,946	31,316	0,000	0,000	3,634
Starch Ø	0,000	25,467	61,337	0,000	0,000	13,197
Starch SD	0,000	36,015	29,593	0,000	0,000	6,580
Stroh Ø	37,809	33,694	7,333	0,000	0,568	20,596
Stroh SD	0,487	0,165	0,475	0,000	0,415	0,551
Sucrose Ø						
Sucrose SD						
Trehalose Ø						
Trehalose SD						
Xylan Ø	38,647	31,334	10,886	5,642	1,625	11,866
Xylan SD	0,322	0,957	0,496	0,498	0,015	0,673
Xylose Ø	33,870	25,886	23,821	4,030	0,000	12,394
Xylose SD	2,648	1,657	4,450	0,594	0,000	0,458

Tab. S8: Produced metabolites from W212 while growing on different c-sources including standard deviation (SD) and excluding ethanol.

	Formate Δ [mmol]	Acetate Δ [mmol]	Lactate Δ [mmol]	Succinate Δ [mmol]	Citrate Δ [mmol]	H2 Δ [mmol]
Cellobiose Ø	1,890	1,191	0,314	0,000	0,000	0,211
Cellobiose SD	0,081	0,058	0,015	0,000	0,000	0,031
Cellulose Ø	1,729	1,413	0,552	0,000	0,000	0,367
Cellulose SD	0,030	0,018	0,042	0,000	0,000	0,024
Fructose Ø	1,803	1,175	0,437	0,000	0,000	0,194
Fructose SD	0,026	0,022	0,057	0,000	0,000	0,016
Glucose Ø	1,901	1,118	0,214	0,000	0,000	0,114
Glucose SD	0,092	0,076	0,031	0,000	0,000	0,002
Inulin Ø						
Inulin SD						
Lactose Ø	0,952	0,784	0,277	0,000	0,000	0,414
Lactose SD	0,020	0,010	0,032	0,000	0,000	0,008
Maltose Ø	1,947	1,145	0,343	0,000	0,000	0,108
Maltose SD	0,060	0,017	0,020	0,000	0,000	0,009
Mannose Ø						
Mannose SD						
Pectin Ø	0,132	0,113	0,000	0,000	0,000	0,024
Pectin SD	0,008	0,092	0,000	0,000	0,000	0,001
Starch Ø	2,026	1,355	0,401	0,000	0,000	0,151
Starch SD	0,081	0,208	0,020	0,000	0,000	0,018
Stroh Ø	0,871	0,910	0,143	0,000	0,014	0,375
Stroh SD	0,010	0,061	0,007	0,000	0,002	0,012
Sucrose Ø	2,227	1,388	0,234	0,000	0,000	0,129
Sucrose SD	0,026	0,163	0,051	0,000	0,000	0,017
Trehalose Ø						
Trehalose SD						
Xylan Ø	1,293	0,837	0,141	0,000	0,068	0,216
Xylan SD	0,033	0,030	0,002	0,000	0,001	0,012
Xylose Ø	1,581	0,948	0,644	0,000	0,000	0,097
Xylose SD	0,089	0,046	0,067	0,000	0,000	0,020

Tab. S9: Relative produced metabolites from W212 while growing on different c-sources including standard deviation (SD) and excluding ethanol.

	Formate/total [%]	Acetate/total [%]	Lactate/total [%]	Succinate/total [%]	Citrate/total [%]	H2/total [%]
Cellobiose Ø	52,414	33,027	8,696	0,000	0,000	5,864
Cellobiose SD	0,533	0,223	0,217	0,000	0,000	0,840
Cellulose Ø	42,577	34,796	13,597	0,000	0,000	9,030
Cellulose SD	0,712	0,569	0,975	0,000	0,000	0,620
Fructose Ø	49,978	32,562	12,100	0,000	0,000	5,361
Fructose SD	1,146	0,719	1,465	0,000	0,000	0,376
Glucose Ø	56,810	33,384	6,377	0,000	0,000	3,428
Glucose SD	0,745	0,356	0,659	0,000	0,000	0,195
Inulin Ø						
Inulin SD						
Lactose Ø	39,211	32,302	11,409	0,000	0,000	17,078
Lactose SD	0,404	0,829	1,161	0,000	0,000	0,516
Maltose Ø	54,946	32,334	9,675	0,000	0,000	3,045
Maltose SD	0,493	0,478	0,332	0,000	0,000	0,329
Mannose Ø						
Mannose SD						
Pectin Ø	55,941	34,396	0,000	0,000	0,000	9,663
Pectin SD	22,254	25,256	0,000	0,000	0,000	3,002
Starch Ø	51,652	34,262	10,248	0,000	0,000	3,838
Starch SD	1,993	2,783	0,836	0,000	0,000	0,249
Stroh Ø	37,708	39,304	6,185	0,000	0,595	16,208
Stroh SD	0,812	1,419	0,483	0,000	0,120	0,292
Sucrose Ø	56,142	34,787	5,823	0,000	0,000	3,247
Sucrose SD	2,879	2,041	1,003	0,000	0,000	0,340
Trehalose Ø						
Trehalose SD						
Xylan Ø	50,585	32,748	5,524	0,000	2,679	8,464
Xylan SD	0,686	0,961	0,161	0,000	0,018	0,565
Xylose Ø	48,327	28,997	19,722	0,000	0,000	2,954
Xylose SD	0,939	0,606	1,983	0,000	0,000	0,524

Tab. S10: Produced metabolites from SA222 while growing on different c-sources including standard deviation (SD) and excluding ethanol.

	Formate Δ [mmol]	Acetate Δ [mmol]	Lactate Δ [mmol]	Succinate Δ [mmol]	Citrate Δ [mmol]	H2 Δ [mmol]
Cellobiose Ø	1,537	0,883	0,820	0,000	0,000	0,279
Cellobiose SD	0,074	0,081	0,078	0,000	0,000	0,025
Cellulose Ø	0,620	0,375	0,000	0,000	0,000	0,186
Cellulose SD	0,027	0,063	0,000	0,000	0,000	0,022
Fructose Ø	1,534	0,914	0,577	0,068	0,000	0,193
Fructose SD	0,002	0,016	0,041	0,048	0,000	0,017
Glucose Ø	1,451	0,858	0,846	0,031	0,000	0,213
Glucose SD	0,072	0,035	0,058	0,044	0,000	0,027
Inulin Ø						
Inulin SD						
Lactose Ø	0,786	0,589	0,465	0,000	0,000	0,328
Lactose SD	0,008	0,014	0,085	0,000	0,000	0,013
Maltose Ø	1,469	0,881	0,722	0,000	0,000	0,208
Maltose SD	0,040	0,023	0,028	0,000	0,000	0,019
Mannose Ø	1,142	0,729	1,230	0,000	0,000	0,238
Mannose SD	0,024	0,024	0,006	0,000	0,000	0,011
Pectin Ø	0,199	0,162	0,000	0,000	0,005	0,027
Pectin SD	0,023	0,008	0,000	0,000	0,005	0,001
Starch Ø	1,510	0,933	0,772	0,032	0,000	0,275
Starch SD	0,302	0,183	0,210	0,045	0,000	0,017
Stroh Ø	0,735	0,609	0,058	0,000	0,003	0,303
Stroh SD	0,002	0,010	0,006	0,000	0,005	0,013
Sucrose Ø	1,463	0,925	1,025	0,062	0,000	0,245
Sucrose SD	0,075	0,051	0,039	0,044	0,000	0,009
Trehalose Ø	0,000	0,000	0,000	0,000	0,000	0,005
Trehalose SD	0,000	0,000	0,000	0,000	0,000	0,000
Xylan Ø	1,153	0,765	0,317	0,038	0,055	0,212
Xylan SD	0,015	0,005	0,071	0,053	0,002	0,007
Xylose Ø	1,367	0,869	0,860	0,000	0,000	0,260
Xylose SD	0,049	0,022	0,106	0,000	0,000	0,015

Tab. S11: Relative produced metabolites from SA222 while growing on different c-sources including standard deviation (SD) and excluding ethanol.

	Formate/total [%]	Acetate/total [%]	Lactate/total [%]	Succinate/total [%]	Citrate/total [%]	H2/total [%]
Cellobiose Ø	43,707	25,044	23,286	0,000	0,000	7,964
Cellobiose SD	0,606	0,898	1,371	0,000	0,000	0,798
Cellulose Ø	52,734	31,573	0,000	0,000	0,000	15,693
Cellulose SD	2,592	2,248	0,000	0,000	0,000	0,362
Fructose Ø	46,704	27,819	17,557	2,041	0,000	5,879
Fructose SD	1,262	0,523	1,117	1,448	0,000	0,395
Glucose Ø	42,681	25,260	24,890	0,894	0,000	6,275
Glucose SD	1,847	1,019	1,726	1,264	0,000	0,912
Inulin Ø						
Inulin SD						
Lactose Ø	36,272	27,191	21,386	0,000	0,000	15,150
Lactose SD	1,167	1,333	3,425	0,000	0,000	1,001
Maltose Ø	44,801	26,860	22,006	0,000	0,000	6,333
Maltose SD	0,839	0,186	0,566	0,000	0,000	0,477
Mannose Ø	34,192	21,813	36,862	0,000	0,000	7,133
Mannose SD	0,288	0,330	0,583	0,000	0,000	0,200
Pectin Ø	50,517	41,331	0,000	0,000	1,210	6,942
Pectin SD	2,429	2,416	0,000	0,000	1,308	0,442
Starch Ø	42,854	26,495	21,741	0,709	0,000	8,200
Starch SD	1,913	1,135	2,508	1,003	0,000	2,021
Stroh Ø	43,009	35,659	3,396	0,000	0,192	17,744
Stroh SD	0,551	0,175	0,330	0,000	0,271	0,638
Sucrose Ø	39,288	24,845	27,587	1,688	0,000	6,591
Sucrose SD	1,357	0,961	1,567	1,198	0,000	0,141
Trehalose Ø	0,000	0,000	0,000	0,000	0,000	100,000
Trehalose SD	0,000	0,000	0,000	0,000	0,000	0,000
Xylan Ø	45,440	30,167	12,438	1,429	2,154	8,372
Xylan SD	1,297	1,021	2,488	2,021	0,017	0,588
Xylose Ø	40,767	25,908	25,575	0,000	0,000	7,750
Xylose SD	1,723	0,881	2,679	0,000	0,000	0,257

Tab. S12: Produced metabolites from X2152 while growing on different c-sources including standard deviation (SD) and excluding ethanol.

	Formate Δ [mmol]	Acetate Δ [mmol]	Lactate Δ [mmol]	Succinate Δ [mmol]	Citrate Δ [mmol]	H2 Δ [mmol]
Cellobiose Ø	1,971	1,419	0,000	0,177	0,000	0,416
Cellobiose SD	0,038	0,000	0,000	0,123	0,000	0,033
Cellulose Ø	1,562	1,214	0,000	0,242	0,000	0,392
Cellulose SD	0,128	0,070	0,000	0,045	0,000	0,020
Fructose Ø	1,937	1,409	0,000	0,237	0,000	0,282
Fructose SD	0,211	0,073	0,000	0,101	0,000	0,009
Glucose Ø	1,985	1,381	0,000	0,324	0,000	0,236
Glucose SD	0,021	0,091	0,000	0,015	0,000	0,019
Inulin Ø						
Inulin SD						
Lactose Ø	1,115	0,824	0,000	0,098	0,000	0,366
Lactose SD	0,198	0,149	0,000	0,070	0,000	0,026
Maltose Ø	0,000	0,000	0,000	0,000	0,000	0,035
Maltose SD	0,000	0,000	0,000	0,000	0,000	0,002
Mannose Ø						
Mannose SD						
Pectin Ø	0,086	0,302	0,000	0,000	0,000	0,020
Pectin SD	0,061	0,069	0,000	0,000	0,000	0,002
Starch Ø	0,000	0,000	0,000	0,000	0,000	0,008
Starch SD	0,000	0,000	0,000	0,000	0,000	0,001
Stroh Ø	0,919	0,367	0,000	0,000	0,011	0,411
Stroh SD	0,056	0,046	0,000	0,000	0,000	0,017
Sucrose Ø	0,169	0,000	0,027	0,000	0,000	0,050
Sucrose SD	0,004	0,000	0,038	0,000	0,000	0,001
Trehalose Ø	0,000	0,000	0,000	0,000	0,000	0,006
Trehalose SD	0,000	0,000	0,000	0,000	0,000	0,000
Xylan Ø	1,260	0,969	0,000	0,217	0,027	0,309
Xylan SD	0,100	0,116	0,000	0,012	0,010	0,007
Xylose Ø	1,714	1,257	0,000	0,186	0,000	0,321
Xylose SD	0,090	0,073	0,000	0,020	0,000	0,034

Tab. S13: Relative produced metabolites from X2152 while growing on different c-sources including standard deviation (SD) and excluding ethanol.

	Formate/total [%]	Acetate/total [%]	Lactate/total [%]	Succinate/total [%]	Citrate/total [%]	H2/total [%]
Cellobiose Ø	49,501	35,633	0,000	4,399	0,000	10,466
Cellobiose SD	1,610	0,463	0,000	3,040	0,000	0,968
Cellulose Ø	45,748	35,649	0,000	7,103	0,000	11,500
Cellulose SD	1,773	2,192	0,000	1,406	0,000	0,459
Fructose Ø	50,048	36,663	0,000	5,926	0,000	7,363
Fructose SD	1,187	2,529	0,000	2,197	0,000	0,726
Glucose Ø	50,602	35,139	0,000	8,250	0,000	6,010
Glucose SD	1,596	1,556	0,000	0,220	0,000	0,506
Inulin Ø						
Inulin SD						
Lactose Ø	46,374	34,249	0,000	3,622	0,000	15,755
Lactose SD	1,290	0,340	0,000	2,583	0,000	3,135
Maltose Ø	0,000	0,000	0,000	0,000	0,000	100,000
Maltose SD	0,000	0,000	0,000	0,000	0,000	0,000
Mannose Ø						
Mannose SD						
Pectin Ø	20,767	74,228	0,000	0,000	0,000	5,005
Pectin SD	15,261	15,541	0,000	0,000	0,000	1,028
Starch Ø	0,000	0,000	0,000	0,000	0,000	100,000
Starch SD	0,000	0,000	0,000	0,000	0,000	0,000
Stroh Ø	53,787	21,401	0,000	0,000	0,643	24,169
Stroh SD	0,226	1,582	0,000	0,000	0,033	1,780
Sucrose Ø	70,336	0,000	8,678	0,000	0,000	20,986
Sucrose SD	9,324	0,000	12,272	0,000	0,000	2,952
Trehalose Ø	0,000	0,000	0,000	0,000	0,000	100,000
Trehalose SD	0,000	0,000	0,000	0,000	0,000	0,000
Xylan Ø	45,309	34,735	0,000	7,820	0,940	11,196
Xylan SD	0,180	1,443	0,000	0,489	0,294	1,237
Xylose Ø	49,297	36,159	0,000	5,347	0,000	9,197
Xylose SD	0,664	0,141	0,000	0,360	0,000	0,483

Tab. S14: Produced metabolites from A252 while growing on different c-sources including standard deviation (SD) and excluding ethanol.

	Formate Δ [mmol]	Acetate Δ [mmol]	Lactate Δ [mmol]	Succinate Δ [mmol]	Citrate Δ [mmol]	H2 Δ [mmol]
Cellobiose Ø	1,717	1,308	0,590	0,216	0,000	0,603
Cellobiose SD	0,044	0,054	0,057	0,023	0,000	0,044
Cellulose Ø	1,268	1,052	0,321	0,131	0,000	0,466
Cellulose SD	0,078	0,080	0,172	0,024	0,000	0,036
Fructose Ø	1,460	1,199	0,615	0,164	0,000	0,430
Fructose SD	0,082	0,077	0,034	0,023	0,000	0,020
Glucose Ø	1,559	1,211	0,576	0,194	0,000	0,500
Glucose SD	0,077	0,055	0,047	0,007	0,000	0,010
Inulin Ø	0,092	0,165	0,021	0,000	0,000	0,037
Inulin SD	0,130	0,234	0,030	0,000	0,000	0,025
Lactose Ø	1,434	1,307	0,317	0,152	0,000	0,549
Lactose SD	0,020	0,043	0,123	0,009	0,000	0,024
Maltose Ø	1,523	1,436	0,651	0,153	0,000	0,438
Maltose SD	0,025	0,176	0,014	0,007	0,000	0,024
Mannose Ø	1,362	1,157	0,508	0,148	0,000	0,323
Mannose SD	0,042	0,168	0,046	0,011	0,000	0,047
Pectin Ø	0,092	0,214	0,049	0,000	0,069	0,020
Pectin SD	0,066	0,185	0,035	0,000	0,002	0,004
Starch Ø	1,501	1,338	0,535	0,168	0,000	0,491
Starch SD	0,144	0,130	0,071	0,011	0,000	0,065
Stroh Ø	0,902	1,054	0,086	0,084	0,013	0,543
Stroh SD	0,008	0,063	0,016	0,008	0,000	0,012
Sucrose Ø	1,611	1,343	0,666	0,194	0,000	0,494
Sucrose SD	0,048	0,067	0,085	0,000	0,000	0,039
Trehalose Ø	0,000	0,000	0,000	0,000	0,000	0,007
Trehalose SD	0,000	0,000	0,000	0,000	0,000	0,005
Xylan Ø	1,325	0,982	0,298	0,124	0,074	0,398
Xylan SD	0,176	0,154	0,063	0,015	0,015	0,011
Xylose Ø	1,405	1,081	0,549	0,187	0,000	0,442
Xylose SD	0,153	0,136	0,079	0,019	0,000	0,011

Tab. S15: Relative produced metabolites from A252 while growing on different c-sources including standard deviation (SD) and excluding ethanol.

	Formate/total [%]	Acetate/total [%]	Lactate/total [%]	Succinate/total [%]	Citrate/total [%]	H2/total [%]
Cellobiose Ø	38,717	29,499	13,315	4,871	0,000	13,599
Cellobiose SD	0,424	0,908	1,453	0,582	0,000	0,779
Cellulose Ø	39,318	32,561	9,489	4,008	0,000	14,624
Cellulose SD	1,631	0,791	4,454	0,335	0,000	2,571
Fructose Ø	37,725	30,991	15,899	4,221	0,000	11,164
Fructose SD	0,342	0,899	0,571	0,441	0,000	1,011
Glucose Ø	38,581	29,981	14,239	4,801	0,000	12,397
Glucose SD	0,571	0,871	0,890	0,293	0,000	0,652
Inulin Ø	10,128	18,220	2,330	0,000	0,000	69,322
Inulin SD	14,324	25,767	3,295	0,000	0,000	43,386
Lactose Ø	38,209	34,785	8,324	4,037	0,000	14,646
Lactose SD	1,377	0,455	2,886	0,190	0,000	1,151
Maltose Ø	36,309	34,061	15,549	3,668	0,000	10,412
Maltose SD	1,478	2,686	1,012	0,320	0,000	0,137
Mannose Ø	39,075	32,927	14,505	4,281	0,000	9,210
Mannose SD	1,998	2,733	0,566	0,614	0,000	0,760
Pectin Ø	29,110	38,030	9,245	0,000	18,005	5,609
Pectin SD	24,858	30,234	6,750	0,000	7,740	3,403
Starch Ø	37,187	33,251	13,251	4,179	0,000	12,132
Starch SD	1,079	3,000	1,197	0,395	0,000	0,674
Stroh Ø	33,649	39,274	3,198	3,129	0,480	20,271
Stroh SD	0,864	1,195	0,604	0,216	0,024	0,565
Sucrose Ø	37,420	31,154	15,430	4,510	0,000	11,486
Sucrose SD	1,162	0,728	1,665	0,136	0,000	1,155
Trehalose Ø	0,000	0,000	0,000	0,000	0,000	100,000
Trehalose SD	0,000	0,000	0,000	0,000	0,000	0,000
Xylan Ø	41,392	30,587	9,218	3,880	2,284	12,639
Xylan SD	0,689	0,885	0,766	0,166	0,163	1,665
Xylose Ø	38,338	29,441	14,927	5,112	0,000	12,183
Xylose SD	0,047	0,598	0,643	0,087	0,000	1,096
