# Supplementary figures and images for: Isolation and Biochemical Characterization of Six Anaerobic Fungal Strains from Zoo Animal Feces
Source: Microorganisms. 2021 Aug 3;9(8):1655. doi: 10.3390/microorganisms9081655 (PMC8399178; doi:10.3390/microorganisms9081655)

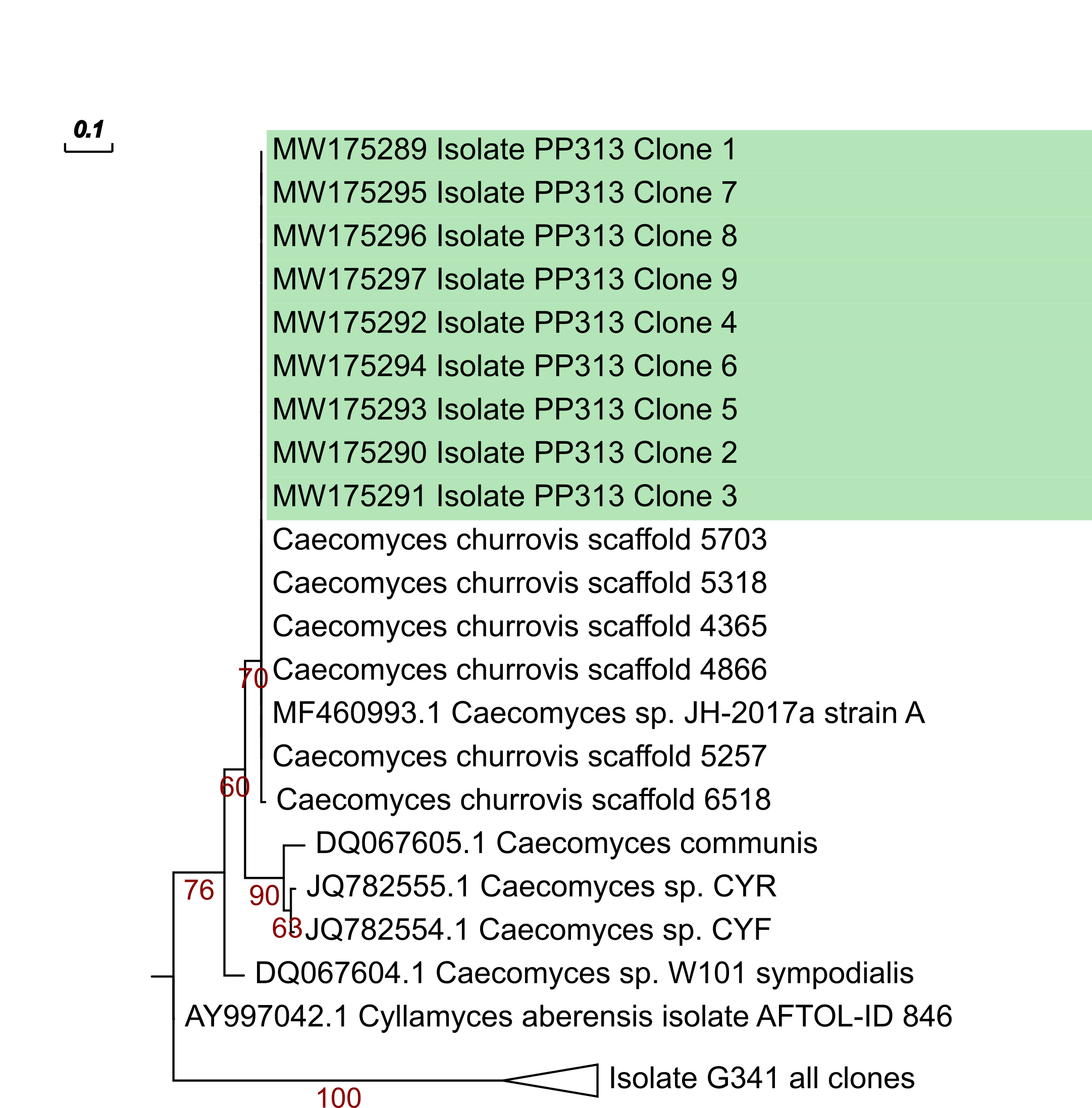

Supplement: Supplementary file 1 [file microorganisms-09-01655-s001.zip › Figure S1.png]
